# Quantifying Public Interest in Police Reforms by Mining Internet Search Data Following George Floyd’s Death

**DOI:** 10.2196/22574

**Published:** 2020-10-21

**Authors:** John W Ayers, Benjamin M Althouse, Adam Poliak, Eric C Leas, Alicia L Nobles, Mark Dredze, Davey Smith

**Affiliations:** 1 University of California San Diego La Jolla, CA United States; 2 Institute for Disease Modeling Bill and Melinda Gates Foundation Seattle, WA United States; 3 Barnard College Columbia University New York, NY United States; 4 Johns Hopkins University Baltimore, MD United States

**Keywords:** policing, digital health, bioinformatics, public health, public interest, data mining, internet, search, trend, Google Trends

## Abstract

**Background:**

The death of George Floyd while in police custody has resurfaced serious questions about police conduct that result in the deaths of unarmed persons.

**Objective:**

Data-driven strategies that identify and prioritize the public’s needs may engender a public health response to improve policing. We assessed how internet searches indicative of interest in police reform changed after Mr Floyd’s death.

**Methods:**

We monitored daily Google searches (per 10 million total searches) that included the terms “police” and “reform(s)” (eg, “reform the police,” “best police reforms,” etc) originating from the United States between January 1, 2010, through July 5, 2020. We also monitored searches containing the term “police” with “training,” “union(s),” “militarization,” or “immunity” as markers of interest in the corresponding reform topics.

**Results:**

The 41 days following Mr Floyd’s death corresponded with the greatest number of police “reform(s)” searches ever recorded, with 1,350,000 total searches nationally. Searches increased significantly in all 50 states and Washington DC. By reform topic, nationally there were 1,220,000 total searches for “police” and “union(s)”; 820,000 for “training”; 360,000 for “immunity”; and 72,000 for “militarization.” In terms of searches for all policy topics by state, 33 states searched the most for “training,” 16 for “union(s),” and 2 for “immunity.” States typically in the southeast had fewer queries related to any police reform topic than other states. States that had a greater percentage of votes for President Donald Trump during the 2016 election searched more often for police “union(s)” while states favoring Secretary Hillary Clinton searched more for police “training.”

**Conclusions:**

The United States is at a historical juncture, with record interest in topics related to police reform with variability in search terms across states. Policy makers can respond to searches by considering the policies their constituencies are searching for online, notably police training and unions. Public health leaders can respond by engaging in the subject of policing and advocating for evidence-based policy reforms.

## Introduction

The death of George Floyd at the hands of police has resurfaced serious questions about police conduct in the United States that has resulted in 1001 deaths of unarmed persons from 2013 through 2019 [1]. Despite widespread protests, public interest in police reform following George Floyd’s death has not been quantified.

Data-driven strategies that identify and prioritize the needs of the public may engender a public health response to improve policing [2,3]. We argue tracking changes in aggregate internet searches is one such strategy, where the content of searches reflects the thoughts and behaviors of the public, the volume of searches reflect their priority, and the location of the search reflects the community [4-6]. This strategy has informed rapid public health responses across many domains, including responses to demand for unproven therapies during the COVID-19 pandemic [7], demand for HIV testing following Charlie Sheen’s public HIV+ disclosure [8], interest in sexual harassment prevention and training following the #MeToo movement [9], and increased suicidal ideation during the initial airing of Netflix’s 13 Reasons Why drama [10], to name a few. Moreover, once traditional data became available—in some cases years later—these example findings were confirmed [11,12], underscoring the validity of mining search histories. Similarly, public health leaders can survey internet search trends to rapidly detect public interest in and priorities for police reform.

Herein, we assess public interest in police reforms by mining aggregate internet searches and discovering the types of reforms being searched for by the public, both on the national and US state level (including Washington DC). Such reforms include police training, police unions, police militarization, and the qualified immunity doctrine that reduces the liability police officers face for potential misconduct [13]. To translate these trends into potential policy making, we quantified the police reforms most searched for by each state (including DC) and how each state’s political culture reflected by results from the 2016 presidential election explained differences in searches.

## Methods

We monitored the fraction of daily Google searches (per 10 million searches) that included the terms “police” and “reform(s)” originating from the United States between January 1, 2010, through July 5, 2020. This strategy would capture “reform the police,” “best police reforms,” etc as markers for general interest in policing reforms. We also monitored searches containing the term “police” with “training,” “union(s),” “militarization,” or “immunity.” These searches were selected because they have been frequently cited by experts as avenues for reforms [14-16], and represent public prioritization of specific policing reform topics. Search rates were monitored both at the national and state (including DC) level.

### Quantifying the Impact of Mr Floyd’s Death

Using historical search rates for each outcome (“police,” “reform[s],” “training,” “union[s],” “militarization,” or “immunity”), we forecasted a counterfactual scenario of expected search rates had Mr Floyd’s death not occurred on May 25, 2020. The expected search rates were forecasted using an autoregressive integrated moving average (ARIMA) model, selected by Hyndman and Khandakar’s algorithm [17], fit to the historical search rates using all available data January 1, 2010, through May 24, 2020. The forecasted search rates were compared to observed search rates from May 25, 2020, through July 5, 2020, including computing the ratio of observed and expected search rates with bootstrap confidence intervals by day and cumulatively for the entire post period.

### Do Search Rates for Specific Police Reform Topics Differ Across States?

First, to assess which states had the greatest number of searches for any policy topic, we estimated the cumulative search rates by state for each policy topic, by summing the fraction of searches per 10 million for the entire post period (May 25, 2020, through July 5, 2020) and ranking states accordingly. Second, to identify which specific policy topics had the greater interest within each state, we estimated each state's total searches for any reform topic, by scaling the cumulative search volumes for each specific policy topic by the total cumulative search volume for all policy topics and ranking states accordingly. For each search query outcome, we contrasted any single state with the mean for all states and compared any two single or group of states by these percentages.

### Does Political Culture Impact Searches for Police Reform Policy Topics?

Given the political implications in policy making, we evaluated how political culture impacted searches for specific policy topics, by comparing cumulative search rates by state for each policy topic against their percent vote share for President Donald Trump during the 2016 election. While crude, the presidential percent vote share is often used as an indicator of the political culture of states, reflecting the strength of support or opposition to conservative or liberal policies [18]. For each, we estimated the breakdown of searches for police reform policy topics by states voting for Hillary Clinton or Donald Trump to evaluate any absolute relationships. Secondarily, we evaluated how more support of Trump predicts searches for police reform policy topics by plotting states by their search volume post Mr Floyd’s death and percent vote share for Trump, and estimated the best fitting line including the slope of this line. All analyses were performed using R, version 3.5.1 (The R Project for Statistical Computing).

## Results

### Internet Searches for Police “Reform(s)”

**Figure 1:**
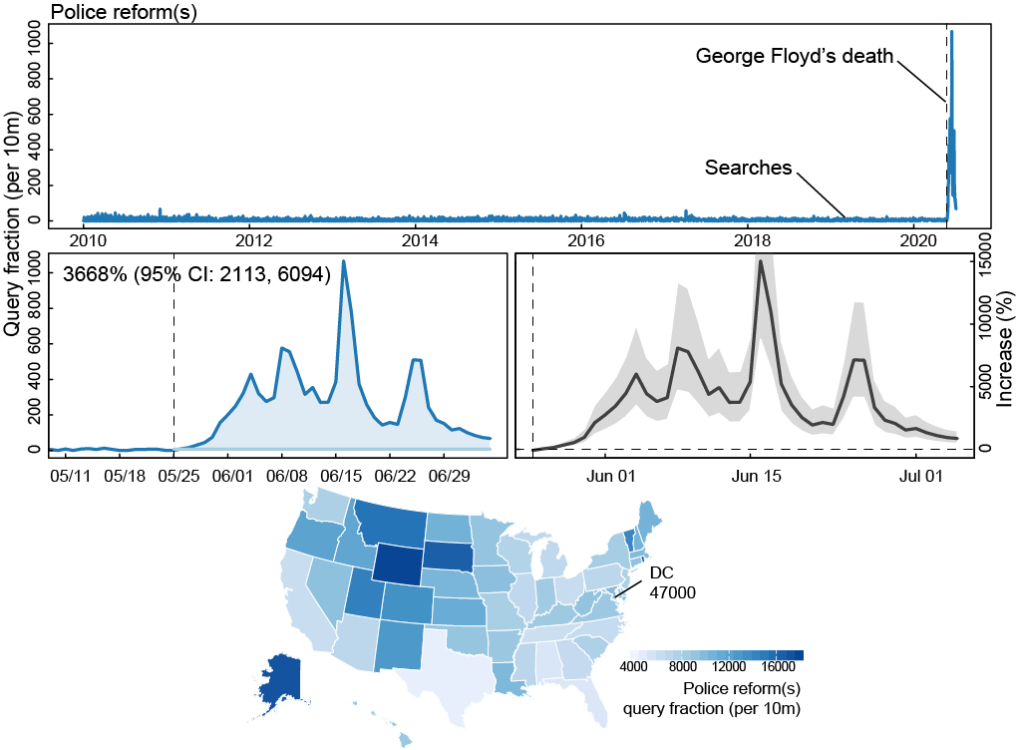
Internet searches for “police” and “reform(s)” following the death of George Floyd. Queries included all searches with the terms “police” and “reform(s).” Top panel shows the historic trends of reform searches in comparison to after Mr Floyd’s death. Middle panels show the percent increases in searches after Mr Floyd’s death (vertical dashed line is the day of the killing, May 25, 2020), and the bottom map shows the cumulative query fraction by state, including Washington DC, for the entire period following Mr Floyd’s death (May 25, 2020, through July 5, 2020).

The 41 days following Mr Floyd’s death corresponded with the greatest number of police “reform(s)” searches ever recorded, with the highest daily fraction of all searches for “reform(s)” eclipsing the past high by over 150-fold (Figure 1). In absolute terms, this translated into about 1,350,000 total searches for police “reform(s).”

Police “reform(s)” search rates cumulatively increased 3668% (95% CI 2113-6094) above expected levels and began increasing on May 25 with queries first eclipsing the past all-time high 5 days later (May 30) and ultimately setting the new record on June 16. During this time, local protests erupted (May 26), police officer Derek Chauvin was charged with third-degree murder and second-degree manslaughter over Mr Floyd's death (May 29), protests went national (May 31), Mr Floyd's brother testified at a House Judiciary Committee (June 10), Mr Rayshard Brooks’ death was recorded by video while struggling with police (June 12), and protests reached their peak (June 14) [19]. While queries declined, they remained significantly above expected levels through the last day of observation (July 5).

All states and Washington DC had statistically significant increases in search rates for police “reform(s).” By total volume, DC led all states with a total query fraction [QF] of 7300 per 10 million searches during the entire period after Mr Floyd’s death, more than 4.2-fold greater than the average for the states. DC was followed by Alaska (QF=2760, 1.7-fold higher), Vermont (QF=2670, 1.65-fold higher), and Delaware (QF=2640, 1.64-fold higher). The states with the lowest cumulative search rates included Arkansas (QF=783, 55% below the mean), Maine (QF=1011, 41% below the mean), Kentucky (QF=1040, 40% below the mean), and Louisiana (1160, 33% below the mean).

### Internet Searches for Specific Police Reform Topics

**Figure 2:**
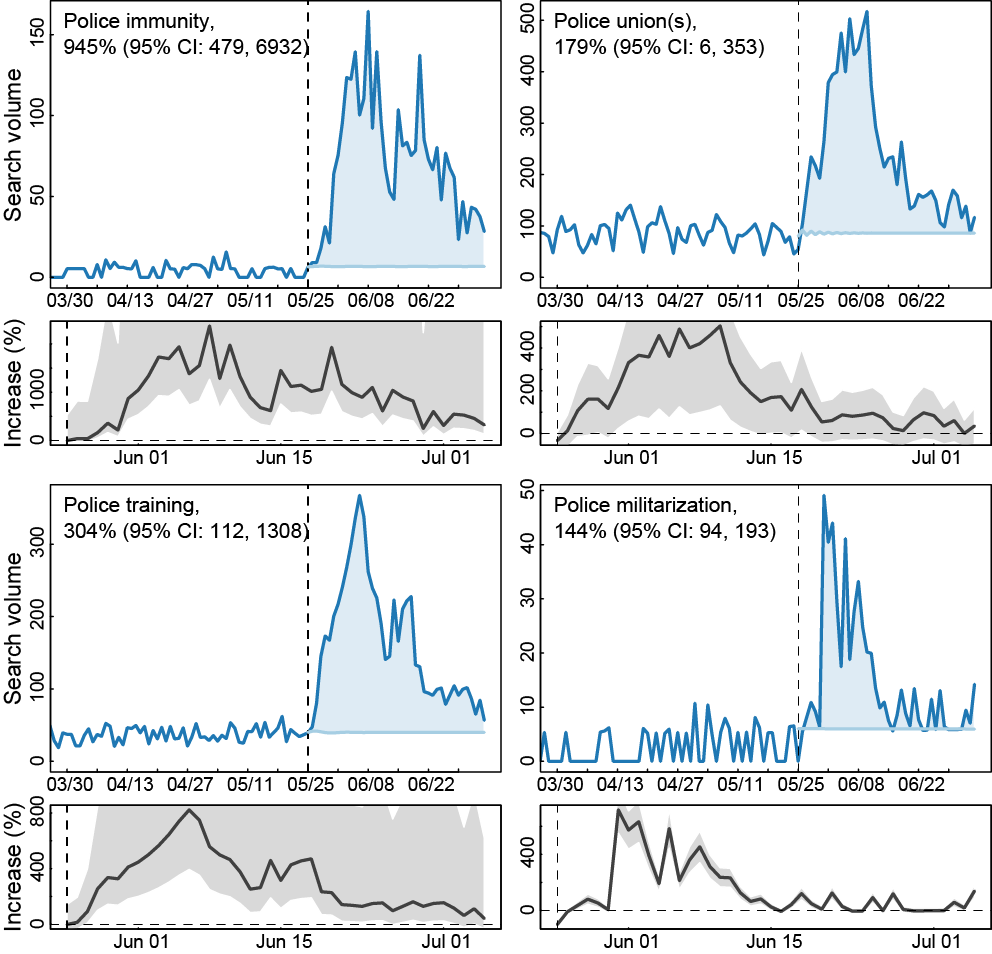
Internet searches for specific police reforms following the death of George Floyd. Queries included all searches with the terms “police” and (A) “immunity,” (B) “union(s),” (C) “training,” or (D) “militarization.” The vertical dashed line corresponds to Mr Floyd's death on May 25, 2020.

Searches for specific reform topics also set new national benchmarks (Figure 2). Searches for “police” and “union(s)” eclipsed the past all-time highs by 4.5-fold, “training” by 4.8-fold, “immunity” by 53-fold, and “militarization” by 34-fold. Statistically, police “immunity” search rates were cumulatively 945% (95% CI 480-6932) higher than expected during the entire post period, followed by “training” (305%; 95% CI 112-1307), “union(s)” (179%; 95% CI 5.8-353), and “militarization” (144%; 95% CI 94-193). This translates into about 1,220,000 total searches for “union(s),” 820,000 for “training,” 360,000 for “immunity,” and 72,000 for “militarization.”

**Figure 3:**
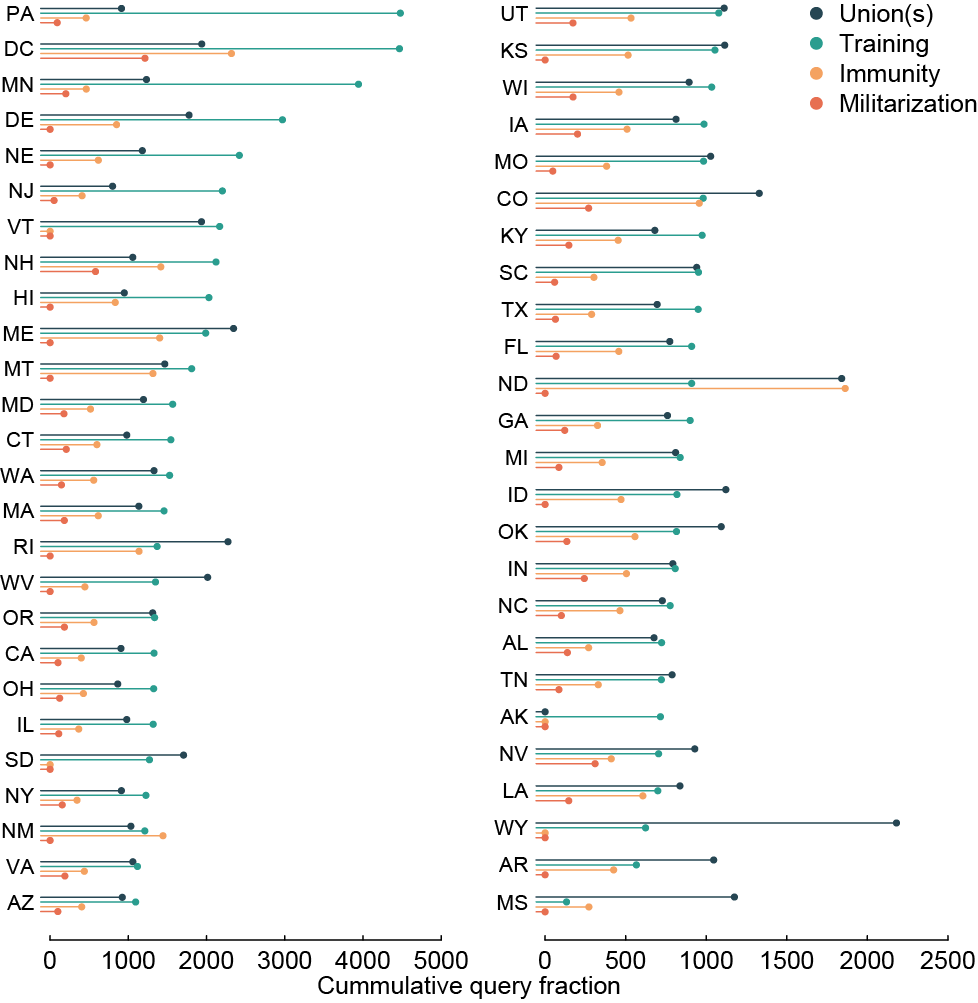
Internet searches for specific police reforms following the death of George Floyd, by US state (including Washington DC). Queries included all searches with the terms “police” and (A) “immunity,” (B) “union(s),” (C) “training,” or (D) “militarization.” The value presented is the cumulative query fraction of searches for the focal terms relative to all searches per 10 million for the period after Mr Floyd’s death (May 25, 2020, through July 5, 2020). States are ordered by cumulative query fraction for “training.”.

By state, all states including DC had statistically significant increases in searches for specific reform topics after the killing of Mr Floyd, including police “union(s)” (led by Maine, Rhode Island, Wyoming, and West Virginia), “training” (led by Pennsylvania, DC, Minnesota, and Delaware), “immunity” (led by DC, North Dakota, New Mexico, and New Hampshire), and “militarization” (led by DC, New Hampshire, Nevada, and Colorado) (Figure 3). Search rates were somewhat correlated by state across categories (overall intraclass correlation coefficient=0.31) and the 5 states with the lowest mean search rate across all outcomes after Mr Floyd’s death were Mississippi, Alabama, Tennessee, Texas, and Arkansas (southern states), and 72% (95% CI 64-84) lower than the 5 states with the highest search rate (DC, Pennsylvania, Minnesota, Maine, and Delaware).

**Figure 4:**
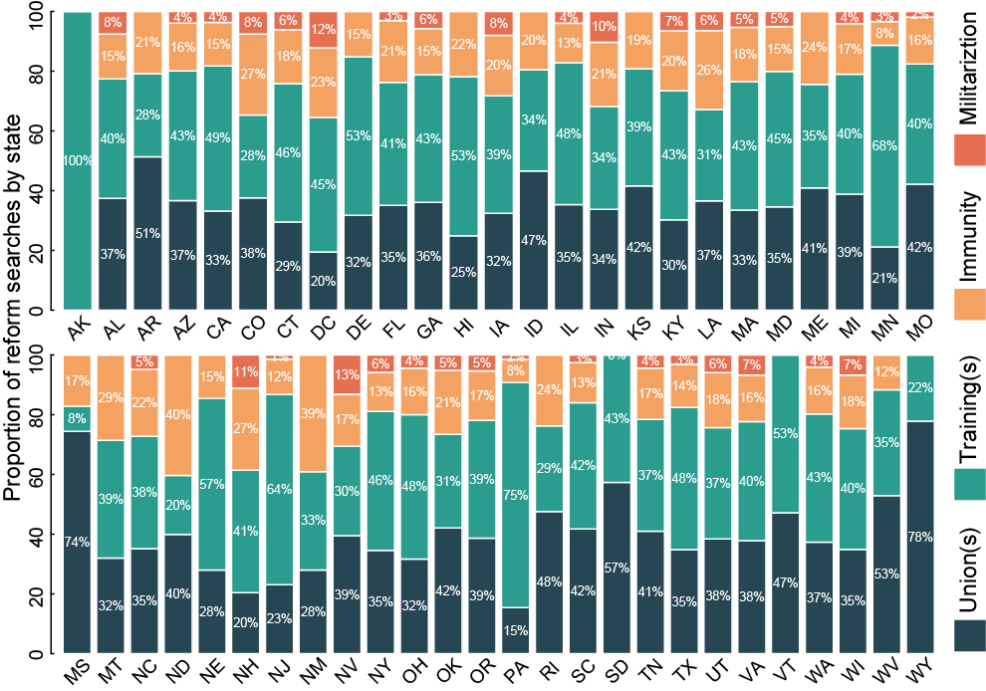
Internet searches for specific police reforms following the death of George Floyd, by US state (including Washington DC). Queries included all searches with the terms “police” and (A) “immunity,” (B) “union(s),” (C) “training,” or (D) “militarization,” normalized to the total volume of searches for all four topics by state following Mr Floyd’s death (May 25, 2020, through July 5, 2020). For instance, 37% for police “union(s)” in Alaska means that 37% of searches for “union(s),” “immunity,” “training,” and “militarization” combined were for “union(s).” States are ordered alphabetically.

Within states, there were typically more searches for police “training” than any other reform topic, followed by police “union(s),” police “immunity,” and police “militarization” (Figure 4). Still, informative differences emerged in how states favored searching for one specific reform topic over another. In total, 33 states searched more often for police “training” than any other policy, including Alaska, Pennsylvania, Minnesota, and New Jersey, averaging 76% of the total search volume for all 4 reform search topics. In 16 states, police “union(s)” was searched more often than any other topic, including Wyoming, Mississippi, South Dakota, and West Virginia, averaging 66%. Only 2 states searched more for police “immunity” (North Dakota and New Mexico), and no states showed increased searches for police “militarization” compared to all other topics.

**Figure 5:**
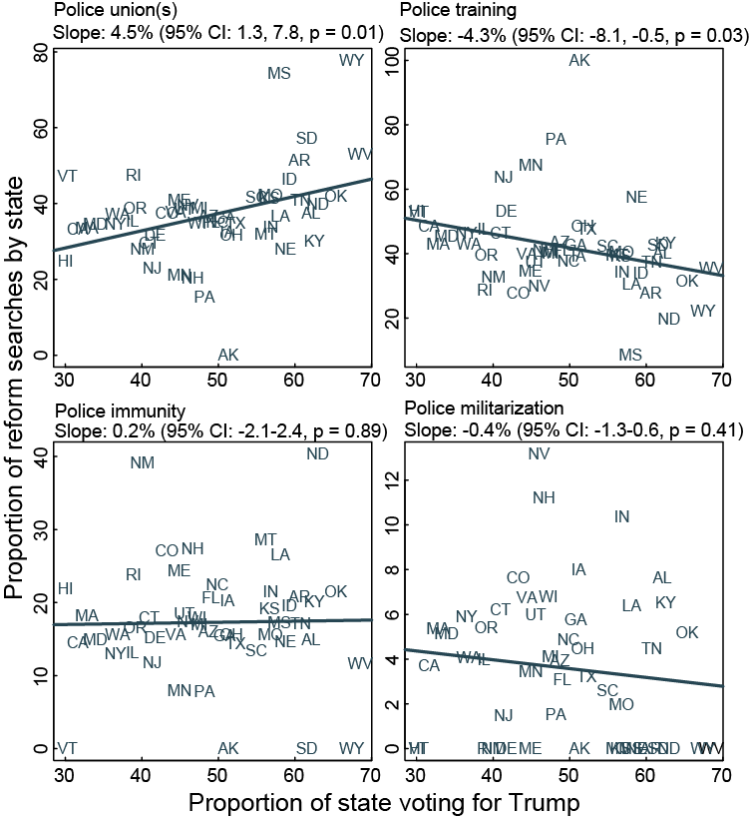
Internet searches for specific police reforms and electoral support for President Donald Trump. Figure shows the proportion of a state voting for Trump in the 2016 election against the proportion of searches for “police” and (A) “immunity,” (B) “union(s),” (C) “training,” or (D) “militarization,” relative to searches for all four reform topics following Mr Floyd’s death (May 25, 2020, through July 5, 2020). Solid lines are linear regressions.

Of the states won by President Trump during the 2016 presidential election, 57% searched more for police “union(s),” while 81% of states won by Secretary Clinton searched more for “training.” Increased vote share for Trump predicted a dose-response relationship with searches for police “union(s)” or ”training” (Figure 5). A 10% increase in vote share for Trump predicted a 4.5% (95% CI 1.3-7.8) increase in cumulative searches for police “union(s)” following Mr Floyd’s death. A similar increase in Trump vote share predicted a 4.3% (95% CI –8.1 to –0.5) decrease in cumulative searches for police “training.” Regardless of a vote preference for Trump or Clinton, states were no more or less likely to search more for police and “immunity” or “militarization” both absolutely and for a marginal change in vote share for Trump.

## Discussion

The United States is at an acute historical juncture with record demand for police reforms sweeping the entire nation. Our results quantify the increasing public interest in police reforms, led by an interest in reforming police training followed by unions, immunity, and militarization nationally. In some states, there is a greater interest in reforming unions or immunity doctrines.

Determining what aspects of police reform to prioritize based on public interest is a challenging problem for elected officials. We argue that monitoring the search queries of a population might clarify the needs and desires of a constituency in real time, especially when little other data is available. The increase in searches related to police reform indicates that the public is actively interested in police reforms, while searches for specific reform topics indicate a desire for leaders to explore these topics. Indeed, many of the reform topics searched for are backed by strong evidence. Restrictions on police unions that potentially advocate for the status quo and hinder reforms and curtailing police immunity that limit criminal and civil liability for police are both effective [20]. On the other hand, some reforms that may be inclusive of searches for “training” are more complex. For example, reforms that implement training on procedural justice or the use of force is associated with less police violence [14], but racial sensitivity and implicit bias training are not associated with less police violence [21,22], even though unarmed Black individuals are more likely to be shot than unarmed White individuals [23]. In these cases, a search for training can serve as the basis for leaders to educate the public and seek additional feedback.

The latter raises a serious limitation of our approach; we cannot link search behavior to a policy preference or identify from whom queries originated. Regardless, searches can predict policy priorities on similar public safety issues, and they overcome many of the limitations that often delay traditional data gathering until the point that the data are no longer actionable [24,25]. Moreover, our focus is restricted to a few reform topics. Additional reform topics, such as body cameras (which have mixed empirical effect) [26] or limiting police funding (with unknown impacts and unanticipated effects) [27] can be studied using our approach. It is important that legislative leaders and policy makers listen to the voices of millions of constituents anonymously searching for solutions and consider policies inclusive of their reform interests as a starting point. Notably, the most high-profile police reform entitled “The Justice Act” proposed by Senator Tim Scott addressed training but did not address police unions, immunity, or militarization. Perhaps amendments to this proposed legislation might consider reforms targeting police unions, given that even in Mr Scott’s home state of South Carolina there were more searches related to police unions than training. Since most policy making occurs among states, decision makers there can rely on our results to identify policies that align with the interests of the public they serve. For instance, in North Dakota or New Mexico, policy makers might consider reforms to police immunity since that reform topic was searched more often than other topics. Extending our work to metropolitan areas can likewise inform more local responses.

While both the American Medical Association [28] and the American Public Health Association [29] have spoken out on police violence, the latter calling it a “public health crisis,” health researchers have done little to advance evidence-based police reforms. As of our writing, only 7 studies on PubMed mention “police” and “misconduct” [30] and 10 mention “police” and “reform(s)” [31] in their title or abstract. This is not to say there is little scientific work on police reform (there is a tremendous literature [32-34]), but little of it has been conducted from a public health perspective or with the benefit of considering strategies popularized by other public health policy successes. For instance, public health advocates have achieved substantial policy reforms to curb deaths attributable to tobacco use [35,36].

Public health scientists, physicians, and other health professionals must do more. Research on policing is essential to public health practice and aligns with public health’s core mission: prevention. The health community has extensive experience studying relevant areas, including unintended medical errors and systematic bias, and can bring those insights to the topic of policing. Moreover, now is the time for health leaders to insist on evidence-based reforms and support science on police reforms that are responsive to the public's needs.

One way to rapidly leverage science to inform policing is to turn to free and abundant big media data. Researchers could further mine internet search data to answer questions related to police practices. For instance, victims of nonfatal instances of police misconduct might search online for help and these digital tracings could be used to evaluate trends and the geographic distribution of misconduct in almost real time. A similar method is already in practice among health researchers who study drug or device adverse events [37,38] and HIV/AIDS, malaria, and tuberculosis in Africa [39]. Expanding the study of policing to other forms of big media data, including social media, online forums, and news media can similarly provide actionable insights.

Policy changes and public health research in police reform lags while public interest in policing reform is at an all-time high. Research that can help inform policies to improve policing should become a priority among the public health community, with our study serving as a demonstration and call to action. By offering to provide policy makers contemporary insights into policing reform topics their constituents have sought online, we hope that policy makers and elected officials will be inspired to act to prevent unnecessary deaths, like Mr Floyd’s.

## Abbreviations

ARIMA: autoregressive integrated moving average
QF: query fraction
